# Evaluation of microbial and vancomycin treatments in ulcerative colitis in murine models

**DOI:** 10.1371/journal.pone.0285613

**Published:** 2023-05-11

**Authors:** Nihal Hasan, Hongyi Yang

**Affiliations:** 1 Department of Clinical Immunology, Tongji Hospital, Tongji Medical College, Huazhong University of Sciences and Technology, Wuhan, People’s Republic of China; 2 Faculty of Health Science, Al-Baath University, Homs, Syria; 3 Department of Microbiology, Northeast Forestry University, Harbin, Heilongjiang, People’s Republic of China; University of Maryland Baltimore, UNITED STATES

## Abstract

**Background:**

Despite the number of available therapies for ulcerative colitis (UC), severe side effects and high cost has limited their clinical application. Thus, finding new alternative strategies with minimal side effects is inevitable. Therefore, this study aimed to compare the effectiveness of different therapeutic approaches in DSS-induced colitis.

**Methods:**

Firstly, we designed oral bio-therapeutic products, Live Bacterial Products (LBP), which include a mixture of fecal bacteria strains isolated from healthy mice and prepared by microencapsulation and freeze-dried techniques. Then we investigated the efficiency of 7 days of freeze-dried FMT, LBP, and vancomycin treatments in DSS-induced colitis. Secondly, we compared the effect of 15 days of microbial therapies (freeze-dried powder of FMT and LBP microcapsules) and seven days of oral vancomycin on the severity of colitis in mice. Furthermore, the levels of IL-1β and TNF-α were measured in serum by ELISA, and the fecal microbiota diversity was analyzed by high-throughput sequencing for all mice groups.

**Results:**

After seven days of treatments, our results indicated that oral vancomycin reduced the severity of DSS-induced colitis in mice, where weight gain and a decrease in IL-1 β and TNF-α levels were observed in the vancomycin group compared with other treatment groups. While after two weeks of treatment, the LBP microcapsules were able to reduce the severity of colitis. And at the end of the treatment period, weight gain and a decrease in the DAI scores and the levels of IL-1β and TNF-α were noted in the LBP treatment group compared to other treatment groups. By high-throughput sequencing of the 16S rRNA gene, our results showed that while the microcapsules LBP treatment increased the fecal microbial diversity, after vancomycin therapy, most of the fecal microbiota genera and operational taxonomic units (OTUs) were depleted.

**Conclusion:**

Our results concluded that treatment duration and preparation methods affect the microbial therapies’ efficiency in UC. Furthermore, this study highlighted the negative consequences of oral vancomycin administration on gut health that should be known before using this medication.

## 1. Introduction

Ulcerative colitis (UC) is one of the chronic inflammatory bowel diseases (IBD) characterized by repeated cycles of relapse and remission. Over the last three decades, increased incidence of IBD in developing countries has become a significant health problem, especially UC which has a high rate of persistent or relapsing symptoms. Several factors are thought to be involved in the UC such as genetic and environmental factors [1], but the exact etiology of UC is still unknown. However, several studies indicated that chronic inflammatory diseases of the gut maybe start with the abnormal interaction of the host immune system and the gut microbiota [2, 3], and this interaction appeared to be fundamental to the pathogenesis of UC [4]. Despite growing evidence of the involvement of the gut microbiota in UC pathogenesis [5, 6], most available treatments target the immune response rather than the colonic microbial environment [7]. Serious side effects and high costs of available therapies still limit their clinical application [8, 9]. Therefore, new treatment methods still need to be investigated. Antibiotics are usually used to prevent and manage infectious complications, while microbial therapies are applied to obtain their total benefits on gut health [4]. Oral vancomycin has been considered an effective medication for IBD treatment [10, 11], compared to traditional antibiotics, vancomycin has a better side effect profile without increasing the risk for vancomycin-resistant enterococcus [12, 13]. However, Ayers and his colleagues indicated that oral vancomycin treatment might be an effective treatment option for IBD in the short and long term [14]. In addition, Tannock et al. reported that oral vancomycin treatment is effective in patients with colitis and suggested a possible relationship between its ability to treat UC and its effect on the vancomycin-sensitive bacteria in the gut microbiota [15], which affect the severity of colitis by attracting neutrophils into damaged colon tissue, thereby to increased colon inflammation [16]. On the other hand, several approaches commonly used to manipulate the gut microbiota have proven high efficiency in IBD treatment [17–20], including prebiotics, fecal microbiota transplantation (FMT) [17–20], and probiotics which have been considered the most popular approach in clinical practice [21]. The manipulation of gut microbiota was considered an attractive therapy for UC due to the ability of the microbiota to reduce the inflammatory capacity of colonized bacteria. Experimentally, probiotics showed anti-inflammatory effects in mice models. During colitis, probiotic provides protective effects by inducing regulatory mechanisms in a strain-specific manner or down-regulating the production of inflammatory cytokines [22]. Several studies on *Lactobacillus* or VSL#3 have proven their efficiency in UC treatment and preventing recurrence [23–25]. Interestingly, using a combination of probiotic strains and prebiotics also showed effectiveness in DSS-induced experimental colitis. For instance, the administration of a mixture of *Lactobacillus acidophilus*, *L*. *plantarum*, *B*. *breve*, and *Bifidobacterium lactis* with inulin improved clinical symptoms and histological scores in DSS-induced experimental colitis and enhanced mucus production in both healthy and DSS-treated mice [26]. Notably, probiotic bacteria strains have shown different effects on the severity of IBD in an experimental model. While *Lactobacillus reuteri* BR11 has reduced the severity of experimental IBD [27], *L*.*crispatus* CCTCC M206119 aggravated it [28]. On the other hand, *L*. *plantarum* NCIMB8826 treatment has led to weight gain and improved colon length without a remarkable effect on the disease activity index and histologic damage in the colitis model [28]. Interestingly, probiotic administration caused a reduction in the expression of pro-inflammatory cytokines and increased intestinal mucin-like and zona occludens-1 expression [29]. Thus, probiotics reduce the damage and barrier dysfunction in UC caused by inflammatory cytokines. Recently, FMT has revolutionized the field of microbial therapeutics. FMT is the transplantation of fecal bacteria from healthy donors to patients with diseases related to microbiota disruption to restore the composition of the normal gut microbiota [30]. Several studies indicated that FMT is an effective treatment for active UC [31–35]. In this context, Colman et al. reported that the clinical remission rate of FMT treatment was 36.2% in IBD patients, including 22% for UC [36]. Another study by Paramsothy et al. indicated that intensive doses of multi-donor FMT stimulated clinical remission and endoscopic improvement in active UC, in addition to causing distinct microbial changes in the gut microbiota composition [37].

This study aimed to investigate the effectiveness of various treatment approaches in experimental colitis models. Therefore, we examined the effect of preparation methods and the treatment duration on the efficiency of microbial treatments compared to vancomycin treatment. Furthermore, we studied the impact of these therapies on the diversity of gut microbiota.

## 2. Materials and methods

### 2.1 Ethics statement

The study was conducted according to the guidelines of the Declaration of Helsinki. The handling of mice and all experimental producers were approved by the Ethics Committee of Northeast Forestry University. All efforts were made to minimize animal suffering.

### 2.2 Bacterial strains

LBP is the mixture of 9 strains of fecal bacteria that were isolated from healthy mice and used for treatment, including *Bacillus* strains *(B*. *velezensis*, *B*. *amyloliquefaciens*, *B*. *cereus*, *B pumilus*, *B*. *siamensis*), *Lactobacillus* strains *(L*. *reuteri*, *L*. *plantarum*, *L*. *johnsonii*), and *Enterococcus faecalis*.

### 2.3 Animals

Fifty male mice (6–8 weeks of age, weighing 20**±**2 g) provided by Northeast Forestry University, Harbin, China, were housed in clean filter-top cages under standard conditions (50%**±**10% humidity, 25°C) in a 12 h dark/12 h light cycles, and feed standard conditions for one week before the experiment.

### 2.4 Antibiotic administration

Mice were treated with vancomycin (500 mg/l; vancomycin hydrochloride, BioFroXX) by solving vancomycin in drinking water.

### 2.5 Induction of acute DSS Colitis

Studies indicated that DSS‑induced acute and chronic colitis showed similar changes in the gut microbiota of the colitis model that was closer to that noticed in UC [38]. For inducing ulcerative colitis, Dextran sulphate sodium (DSS, molecular weight 500,000; MP Aladdin Inc., Shanghai, China) was added to drinking water to the concentration of 5% (w/v) for 7 to10 days, then shifted to normal drinking water.

### 2.6 Preparation of freeze-dried powder

#### 2.6.1 Freeze-dried LBP

Freeze-dried LBP was prepared using the standard methods as previously described by Hamilton et al. [39], by using 10% Difco Skim Milk containing 0.04% L-cysteine. The final product was held at -20°C until used. The total dose was 2.5 ×10^12^ cells. Cell numbers were determined by using poured plate methods. In this study, mice were administrated freeze-dried microbiota powder by oral gavage, 200 μl per dose for 7 days. The daily dose was prepared by adding the freeze-dried powder to PBS (pH 7.4) to the concentration of 10^9^−10^10^ CFU/ml.

#### 2.6.2 Freeze-dried FMT material

Fresh stool samples were collected after 7 days of acclimatization, fecal samples were collected from healthy mice on the 7^th^ day and used for assessment of bacterial isolation, then mixed 1:4 (w/v) with sterile PBS containing 10% sucrose as a cryoprotectan and sieving as described by Hamilton et al. [39], immediately transferred to a freeze-drying device and dried for 24 hours under vacuum at -20°C. The freeze-dried powder was suspended in PBS, prepared serial dilutions that were cultured under aerobic and anaerobic conditions on different media. Cell numbers were determined by poured plats method. The daily dose, used in this study, was prepared as previously described [40] for Freeze-dried bacteria powder.

### 2.7 Preparation of bacterial microcapsules by emulsion method

Bacterial microcapsules were prepared according to Muthukumarasamy et al. [41]. The mixture (50 ml) of cell suspension and sodium alginate was added drop-wise 1:5 (w/v) to vegetable oil containing Tween 80 (0.2%) and stirred for 10 min at 200 rpm by magnetic stirring, then this mixture was injected by a syringe into sterilized 0.2 mol/l CaCl2 solution that was stirred consistently to shaping microcapsules. The microcapsules were left to harden for about 30 min in CaCl2 solution and then washed twice with 0.1% peptone solution to get rid of surplus calcium ions and uncoated cells. The wet alginate beads were flooded in 100 ml of chitosan solution (0.4%, w/v) and shaken at 100 rpm for 40 min on an orbital shaker for coating ensured by rinsing with 0.1% peptone solution to get rid of surplus chitosan. The capsules were frozen at -20°C overnight. The samples were immediately freeze-dried f for 33-h in a freeze-dryer. In this study, mice were administrated freeze-dried microcapsules powder by oral gavage, 200 μl per dose for 2 weeks. The daily dose was prepared by adding the freeze-dried powder to drinking water to a concentration of 10^9^−10^10^ CFU/ml. As described for Freeze dried microcapsules administration another group of mice was administrated freeze dried-microcapsules powder and pectin.

### 2.8 Evaluation of colitis

Colitis was evaluated by disease activity index (DAI), which combined scores of body weight loss, stool consistency, and fecal occult blood (Table 1).

**Table 1:** DAI score.

Fecal consistency	Fecal occult blood	Body weight loss (%)	Score
Normal	Normal	0	0
		1–15	1
Soft	Positive	>5–10	2
		>10–15	3
Diarrhea	Naked eye	>15	4

### 2.9 Detection of levels of IL-1β and TNF-α

At the end of the treatment period, blood samples were collected from all mice groups individually by using heparin as an anticoagulant. Levels of IL-1β and TNF-α in the mice serum were assessed by ELISA Kits (AMEKO, Shanghai, China) according to the manufacturer’s instructions. The differences between IL-1β and TNF-α of different groups were analyzed using Duncan’s multiple range test of SPSS Version 22.0.

### 2.10 Sample collection

Fresh stool samples were sterilely collected from each group of mice every two days during the DSS and treatment period and immediately stored at– 80°C.

### 2.11 DNA extraction and 16S rRNA gene sequencing

DNA extraction was performed with a DNA extraction kit. DNA concentration was measured using NanoDrop 2000. PCR was performed using adapter primers. Sequencing libraries were generated using DNA Library Prep Kit for Illumina following the manufacturer’s recommendations. After cluster generation, the library preparations were sequenced on an Illumina platform and paired-end reads were generated. Raw sequences were transformed into clean reads after data processing. Bioinformatic analysis was performed by QIIME 1.8.0, FLASH 1.2.3, and USEARCH 7.0.

### 2.12 Statistical analysis

Statistical Package for Social Sciences (SPSS) software version 22.0 (SPSS Inc., Chicago, IL, USA) was used to perform statistical analysis. The results are expressed as mean± standard error of the mean (SEM).

## 3. Result

### 3.1 Comparison of microbial therapies and vancomycin after seven days of treatment

#### 3.1.1 Body weight changes

**Fig 1:**
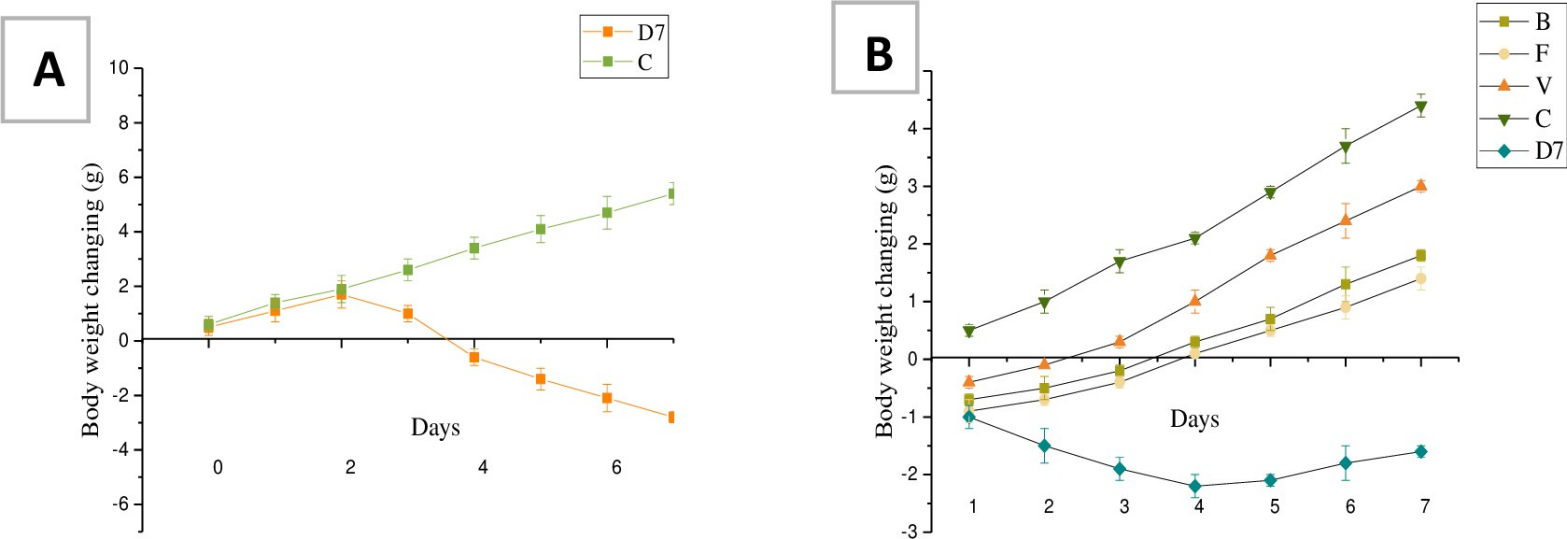
The body weight changes during DSS treatment (A) and treatment period (B). C: Control group, B: a mixture of nine kinds of bacterial freeze-dried powder, V: vancomycin group, F: FMT group, D7: model group.

No significant difference was observed in the body weight of mice among the 5 groups on day 0. Body weight increased gradually in the control group (Fig 1). The body weight of the DSS groups started decreasing on the 3^rd^ day. And the body weight maintained decreasing to 7^th^ days due to inducing experimental colitis.

After removing the DSS solution, the body weight of all mice groups started increasing except for the D7 group. The weight gain of the D7 group was observed after 4 days of lack of DSS (Fig 1). On the 7^th^ day of the treatment period, the difference in the body weight changes for all groups was statistically significant (p < 0.05). The weight gain of the V group was quicker than those of other treatment groups.

#### 3.1.2 Disease activity index scores

**Fig 2:**
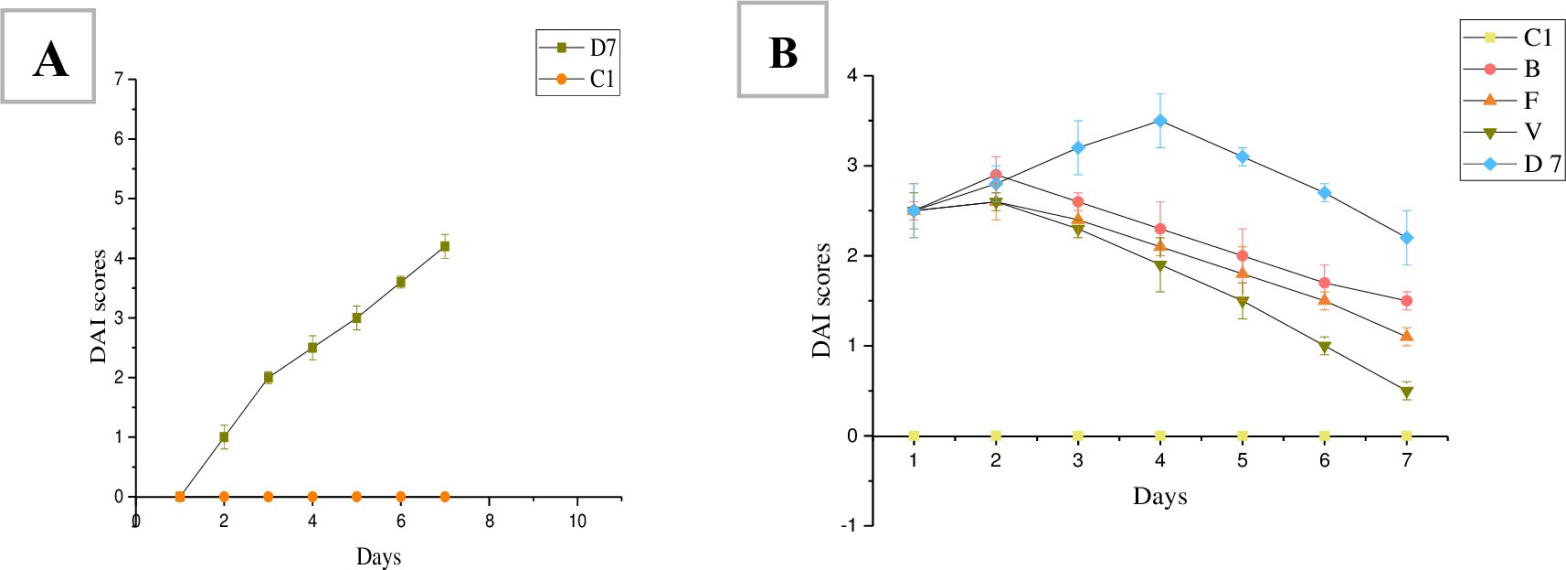
DAI scores during DSS treatment (A) and treatment period (B). C: Control group, B: bacterial powder group, F: FMT group, V: vancomycin group, D 7: model group.

DAI scores were measured during DSS and treatment period (Fig 2). Two days after removing the DSS solution and replaced with tap water, the DAI scores of the three treatment groups (F, B, and V) decreased. The highest DAI score was observed on the 4^th^ day of treatment in the D group. On the 7^th^ day of the treatment period, the DAI score of the V group was lower than those of the B or F group (p < 0.05).

#### 3.1.3 Detection of the level of tumor necrosis factor-α (TNF-α) and interleukin-1β (IL-1β)

**Fig 3:**
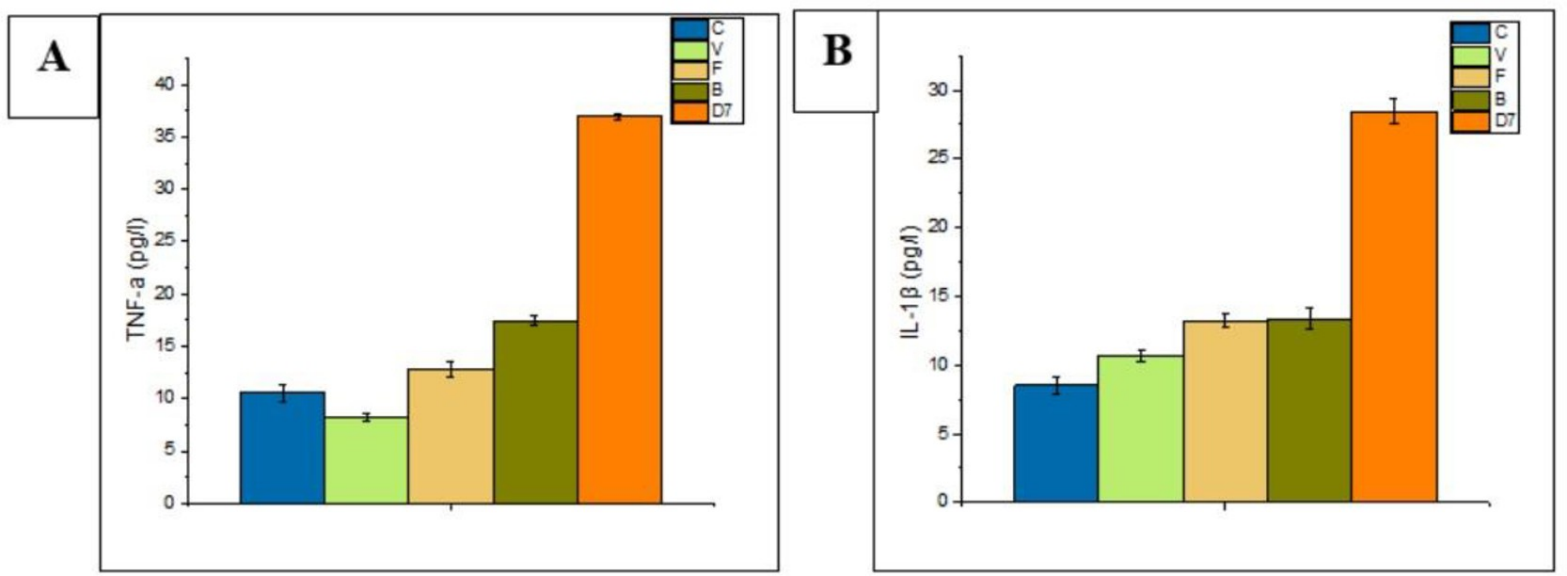
Level of TNF-α and IL-1β based on ELISA analysis. A. The concentration of TNF-α was detected in each group of mice serum. B. The concentration of IL-1β was also detected in each group of mice serum. Data were presented with mean ± SD of three independent experiments.

The concentrations of TNF-α and IL-1β of the three treatment groups (V, F, and B) were lower than that of the D7 group. Among 3 treatment groups (V, F, and B), levels of TNF-α (8.186±0.311 pg/l) and IL-1β (10.718±0.430 pg/l) were lowest in the vancomycin group (Fig 3). In general, all treatment groups (F, B, and V), especially the vancomycin group, showed decreasing in levels of IL-1 β and TNF-α. These results indicate that the three treatments used in this experiment may have reduced the inflammatory condition of mice.

### 3.2 Comparison of microbial therapies and vancomycin after 15 days of treatment

#### 3.2.1 Body weight changes

**Fig 4:**
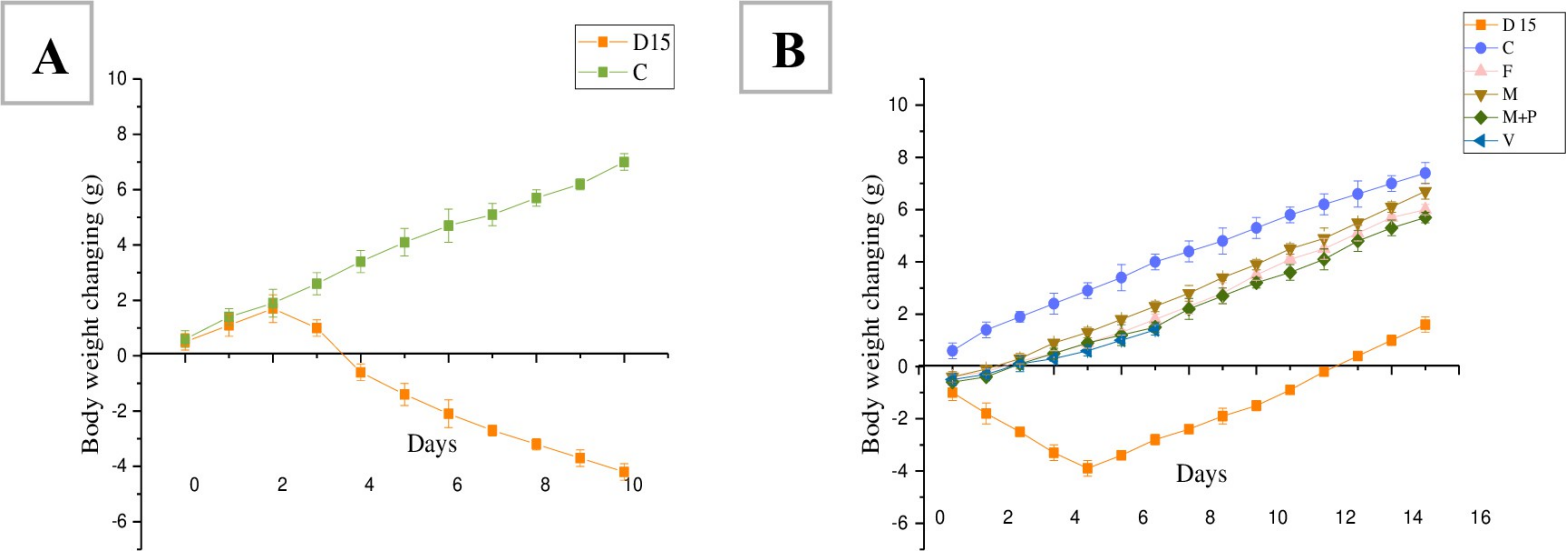
The body weight changes during DSS treatment (A) and treatment period (B). C: Control group, M: bacterial microcapsules group, M+P: bacterial microcapsules+ pectin group, V: vancomycin group, F: FMT group, D15: Model group.

Administration of 5% DSS by drinking water for 10 days was carried out to induce acute colitis in mice. The body weight began decreasing within 3–10 days after DSS administration. The body weight loss for all groups during DSS and treatment period showed in (Fig 4). During DSS administration, no significant difference was observed in the body weight of mice among the 6 groups on day 0. Body weight increased gradually in the control group. The body weight of DSS groups started decreasing on 3^rd^ day. And the body weight maintained decreasing to 10^th^ days due to inducing acute experimental colitis. After removing the DSS solution and shifting to drinking tap water, the body weight of all treatment groups (M, M+P, V, and F) increased while the body weight of mice in the D15 group continued decreasing until 4^th^ day of lack of DSS, the body weight gain was observed in 5^th^ day.

#### 3.2.2 Disease activity index scores

**Fig 5:**
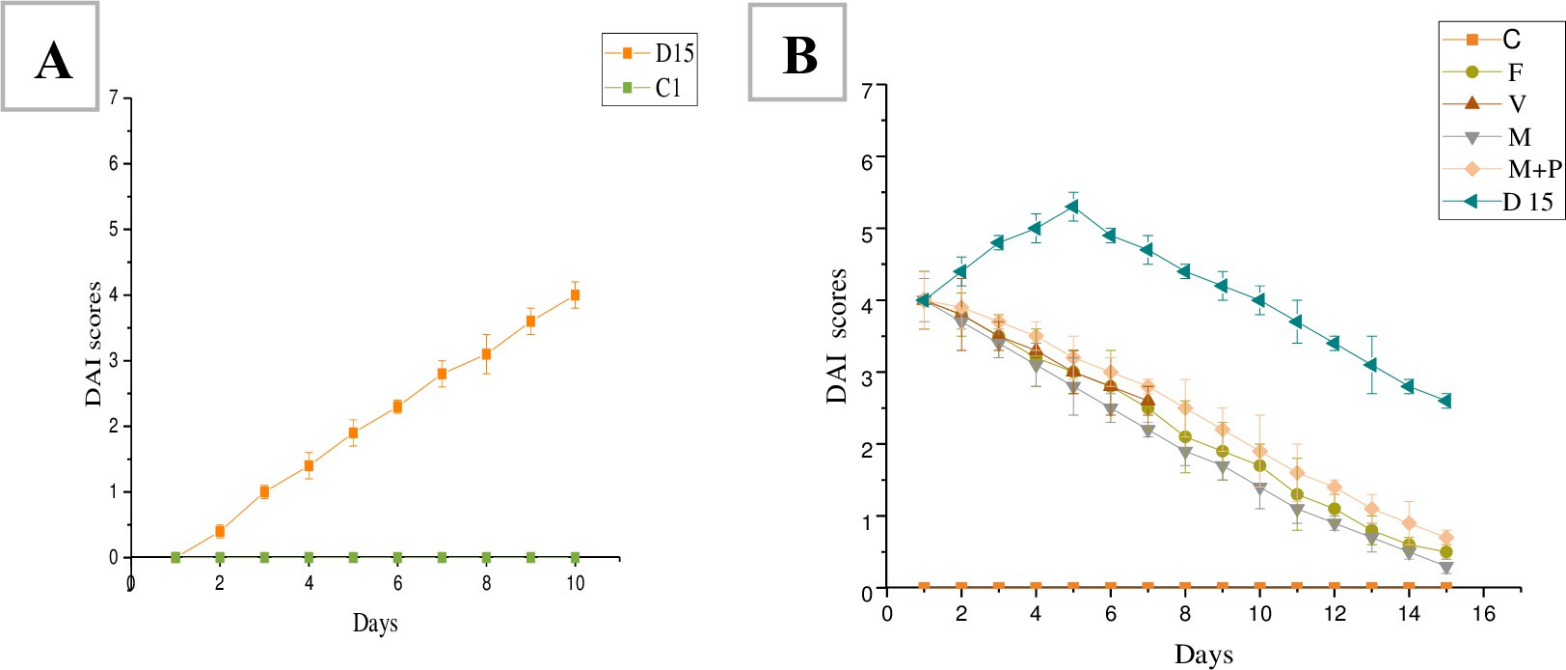
DAI scores during DSS treatment (A) and treatment period (B). C: Control group, M: bacterial microcapsules group, M+P: bacterial microcapsules+ pectin group, V: vancomycin group, F: FMT group, D15: Model group.

DAI scores were measured during DSS and treatment period (Fig 5). After shifting to drinking normal water and starting treatment, the bleeding gradually disappeared, and the body weight increased in all treatment groups. In vancomycin and microcapsules treatment groups, the bleeding disappeared after 1–2 days of treatment, while in the FMT and microcapsules with pectin treatment groups disappeared after 4–6 days. For the D15 group, after shifting to drinking tap water, continued bleeding was observed for five days, and two mice died due to serve colitis, while the body weight gain and the bleeding disappearance were observed after 6–15 days. A high DAI score was observed during DSS administration. After starting treatment, the DAI scores of the treatment groups (M, M+P, F, and V) started decreasing, and the lowest DAI scores were observed in the M group after 15 days of treatment. The DAI score of the D15 group continued to increase and reached the highest peak after five days of removing the DSS solution.

#### 3.2.3 Level of IL-1β and TNF-α

**Fig 6:**
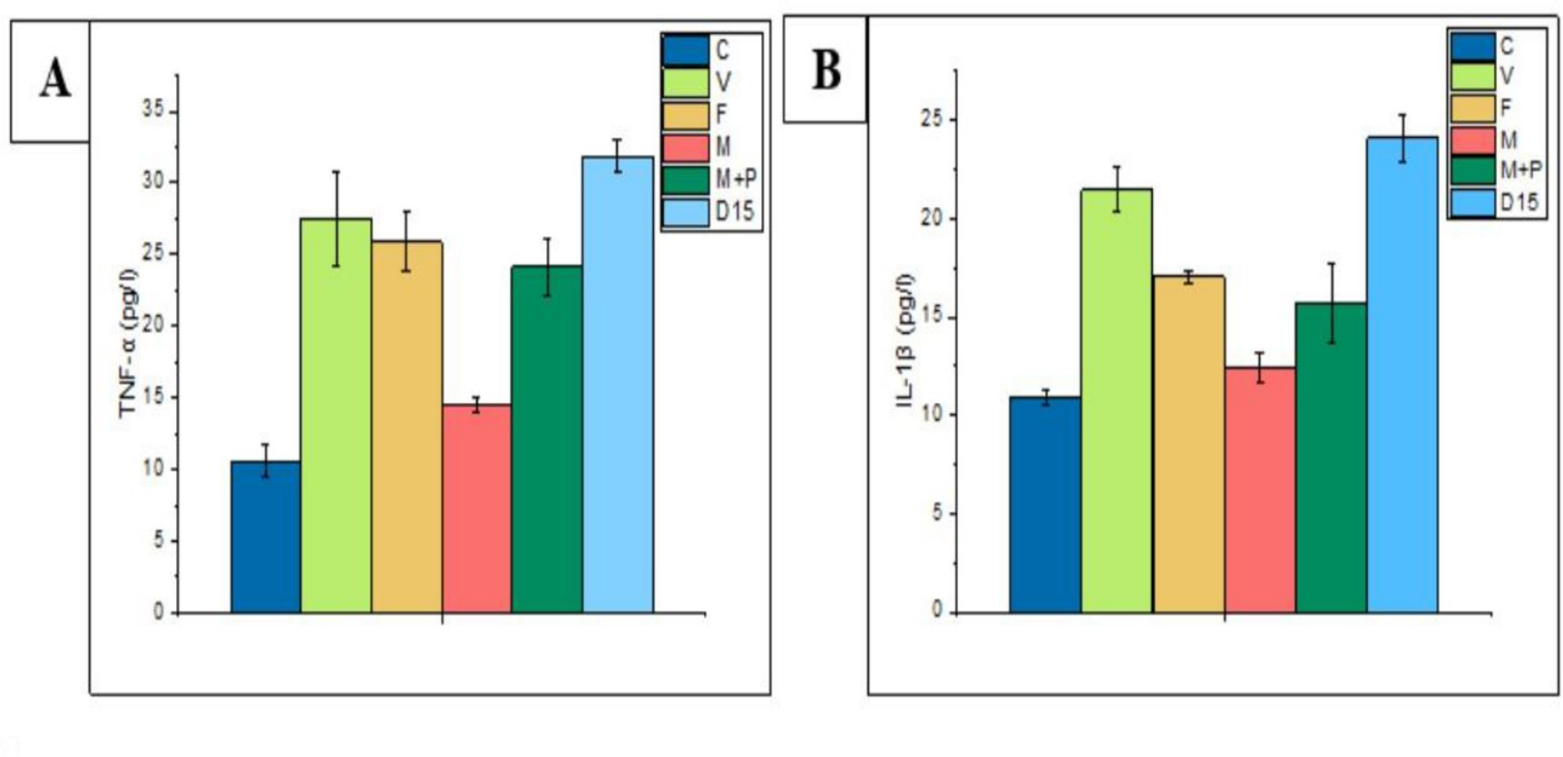
Level of TNF-α and IL-1β based on ELISA analysis. A. The concentration of TNF-α was detected in each group of mice serum. B. The concentration of IL-1β was also detected in each group of mice serum. Data were presented with mean ± SD of three independent experiments.

Generally, levels of IL-1β and TNF-α in all treatment groups were low compared to the D15 group. Our results showed that the highest level of IL-1β (21.480±1.122) was observed in the vancomycin group, while the lowest level (12.401±0.793) was in the microcapsules group (Fig 6). On the other hand, the lowest level of TNF-α (14.580±0.513 pg/l) was observed in the M group, and the highest level of TNF-α (27.496±3.357 pg/l) in vancomycin groups (Fig 6).

### 3.3 Effect of LBP microcapsules and vancomycin treatments on the fecal microbiota diversity

**Fig 7:**
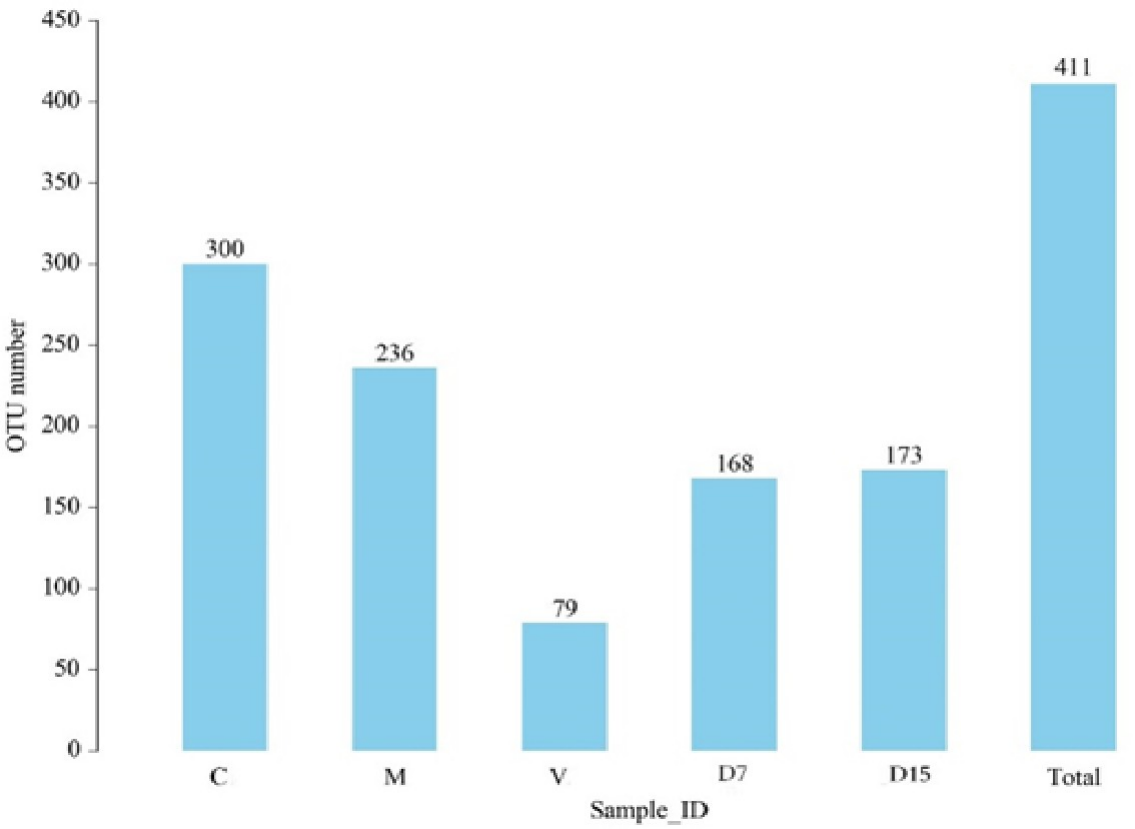
OTUs numbers of different samples. C: normal group sample, M: microcapsules treatment group sample, D7 (DSS treatment for 7 days), D15 (DSS treatment for 15 days), V: vancomycin treatment sample.

We obtained a total of 411 operational taxonomic units (OTUs): between 79 and 300 OTUs for each sample (Fig 7). The highest OTUs number was observed in the control and microcapsules groups, while the vancomycin group showed the lowest OTUs number.

**Fig 8:**
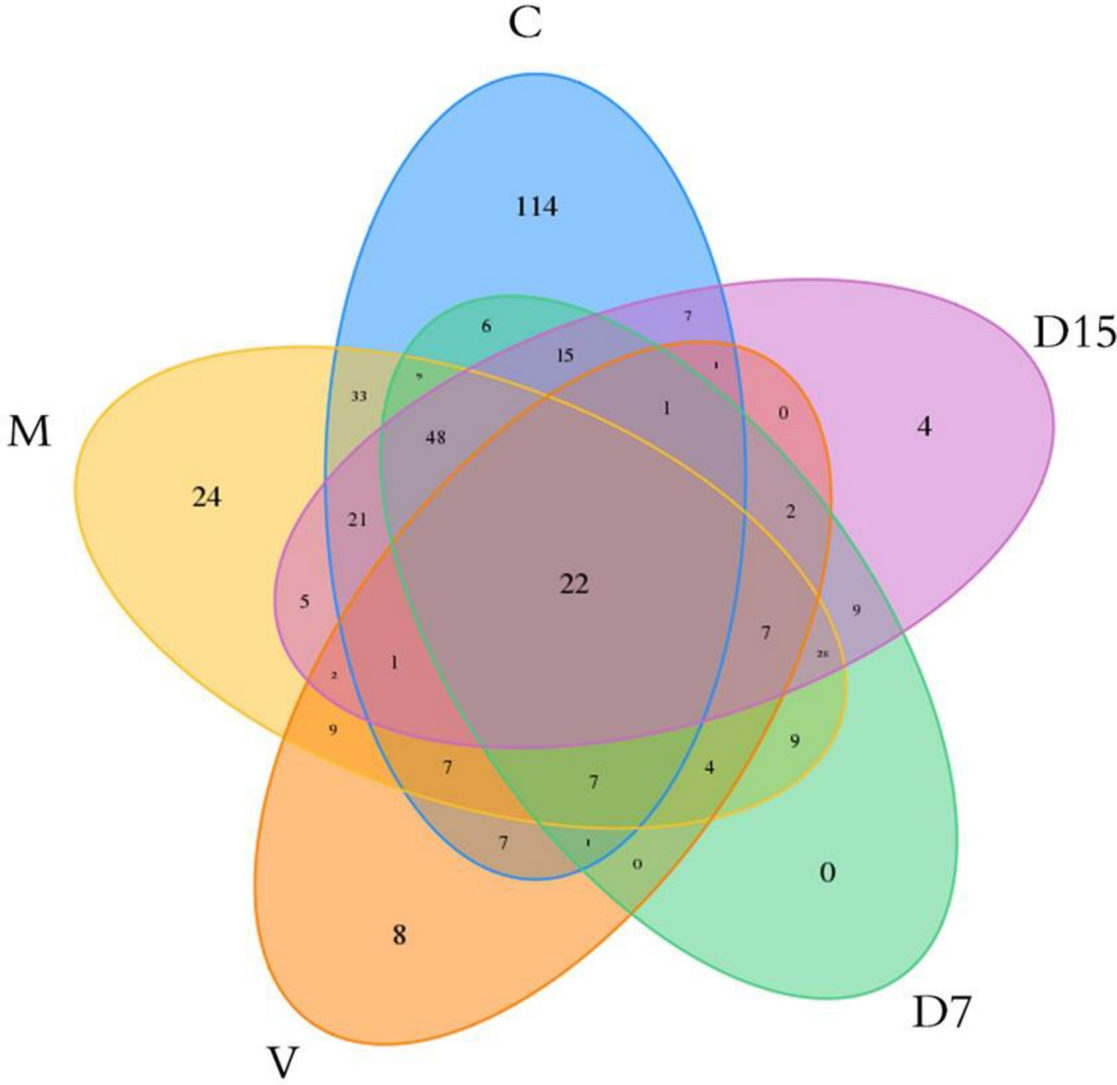
Venn diagram showing the specific and shared OTUs of different groups. C: Control group sample, M: microcapsules treatment group sample, D7 (DSS treatment for 7 days), D15 (DSS treatment for 15 days), V: vancomycin treatment sample.

There were 22 OTUs shared by all groups, and 114, 24, 8, 4, and 0 unique OTUs in the C, M, V, D15, and D7 groups respectively (Fig 8).

Sequences were classified into 9 phyla, 15 classes, 23 orders, 43 families, 127 genera, and 134 species (Table 2). Control group samples showed the highest number of fecal bacterial species, whilst the lowest number was observed in the vancomycin group. The diversity index, including Shannon, Simpson, and community richness- Chao1 and ACE, were shown in (Table 3). The highest microbial diversity was observed in the control and microcapsules groups (3.923, 3.7358 in Shannon, and 0.0467, 0.0506 in Simpson index, respectively). While the lowest diversity was observed in vancomycin groups (1.3789 in Shannon, and 0.4122 in Simpson, respectively). In addition, the highest species richness was observed in the control and microcapsules groups (317.1, 267.625 in Chao1, 318.2715, 279.5385 in ACE for C1 and M samples respectively), and the lowest species richness was observed in the vancomycin group (102.0 in Chao1, 116.2793 in ACE) (Table 3).

**Table 2:** This table indicates the difference in some index related to bacteria diversity.

Sample	Kindom	Phylum	Class	Order	Family	Genus	Species
C	1	9	15	20	38	104	107
D7	1	8	13	19	33	74	77
D15	1	8	12	15	29	71	73
M	1	8	14	22	38	92	97
V	1	8	13	19	32	59	62
Total	1	9	15	23	43	127	134

**Table 3:** The table indicates the classification of bacteria in each sample.

Sample ID	OTU	ACE	Chao1	Simpson	Shannon	Coverage
C	300	318.2715	317.1	0.0467	3.923	0.9998
M	236	279.5385	267.625	0.0506	3.7358	0.9998
V	79	116.2793	102.0	0.4122	1.3789	0.9998
D15	173	197.6587	192.1176	0.0935	2.9064	0.9997
D7	168	194.9129	193.375	0.1523	2.4205	0.9997

**Fig 9:**
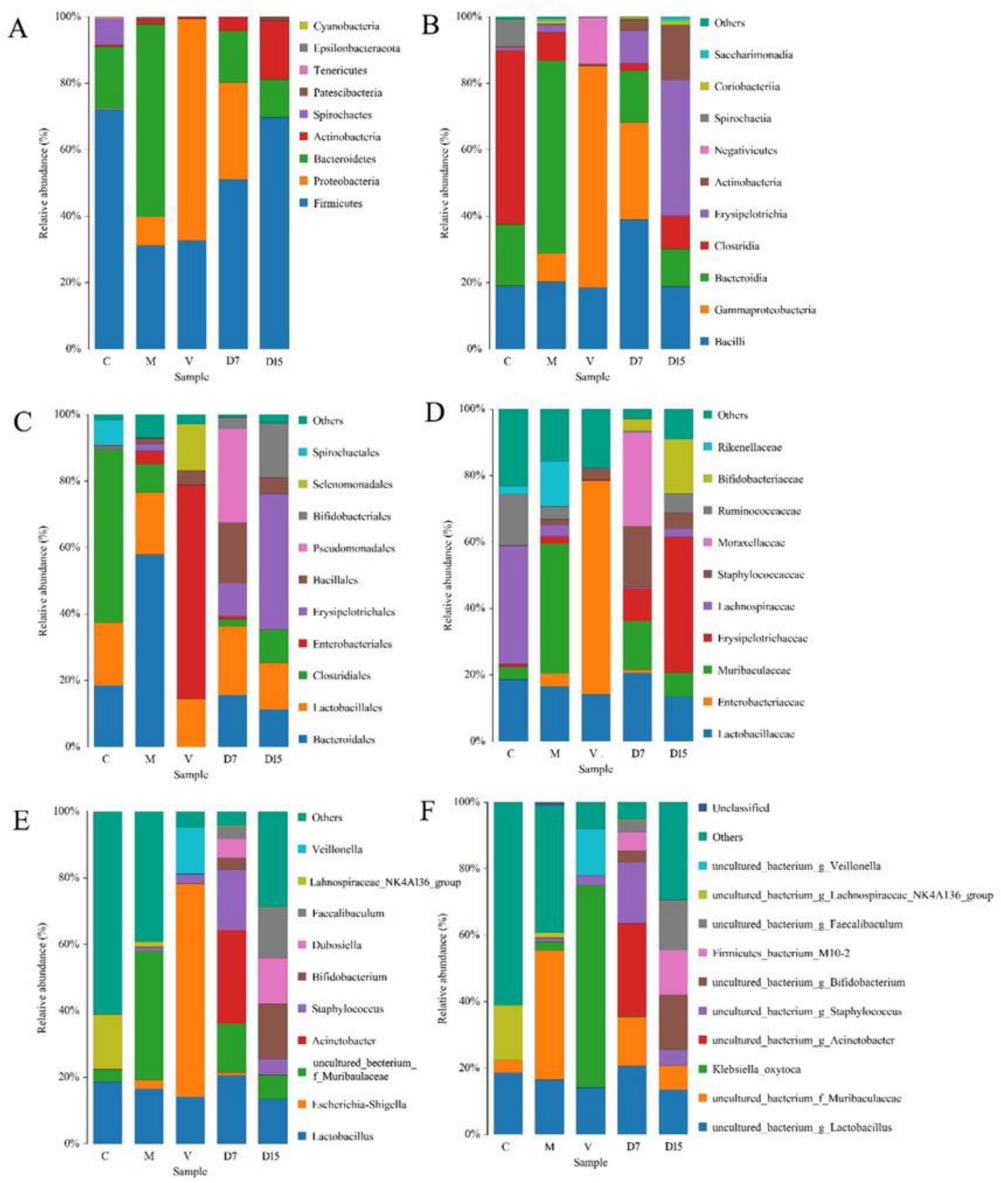
Bar charts showing the taxonomic classification and the relative abundance of fecal microbiota. Phylum, Class, Order, Family, Genus, and Species-level classifications are shown in panels (A–F) respectively.

The characterization of fecal microbiota was a significant difference among the five groups (Fig 9).

Taxonomy data and relative abundance, which were obtained from samples taken from the five groups (C, M, D7, D15, and V), were significantly different. At the phylum level, fecal bacterial communities showed clear differences in the Firmicutes abundances among all groups. The fecal microbiota of the control group (C) and DSS groups (D7, D15) was dominated by Firmicutes, whilst the vancomycin groups and microcapsules group were dominated by Proteobacteria, and Bacteroidetes, respectively. At the genus level, the dominant genera of the vancomycin group were represented by *Escherichia-Shigella*.

### 3.4 Differential analysis of gut microbiota at the general level

**Fig 10:**
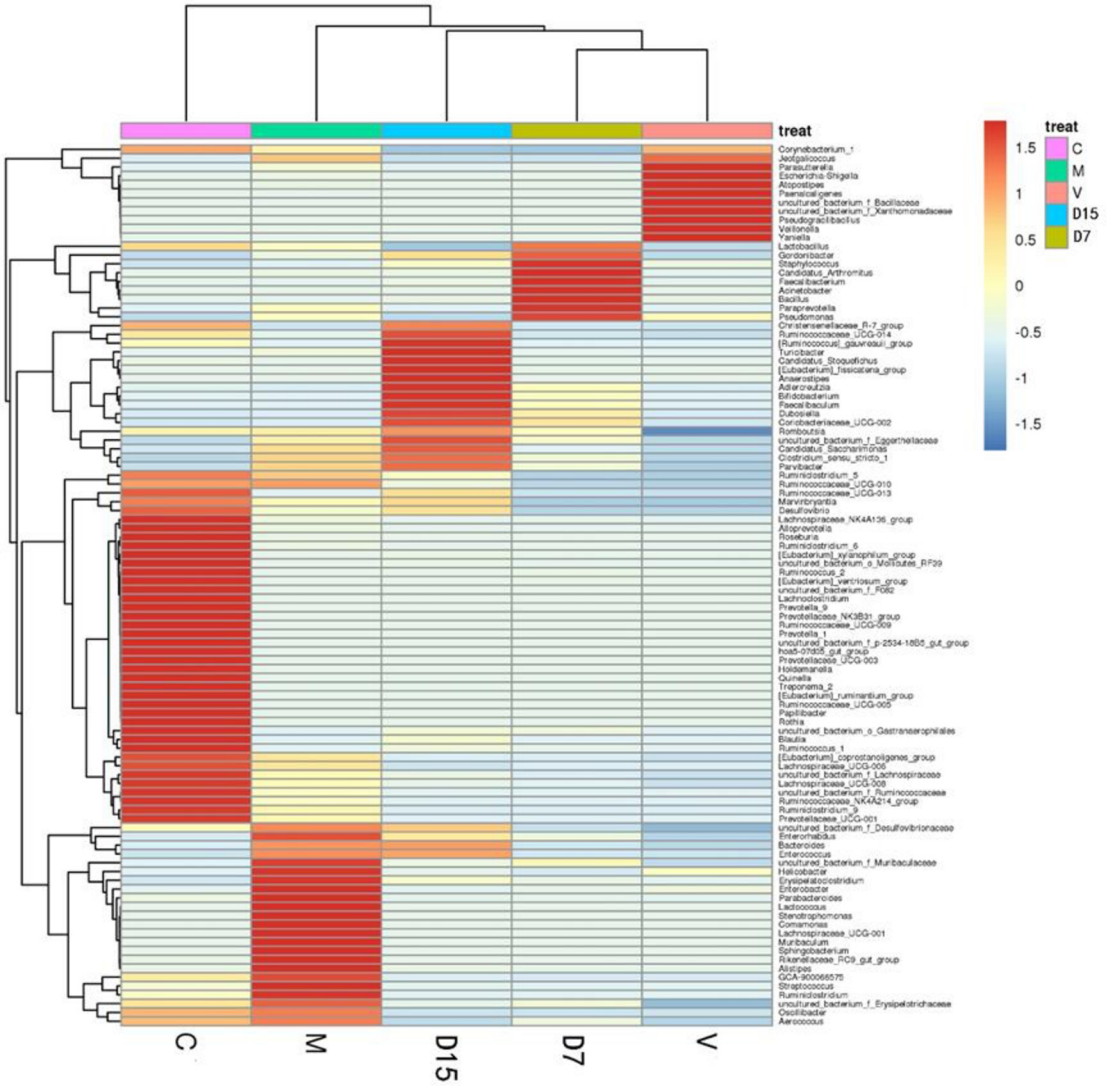
Clustering of different treatment groups according to their microbial structure. Heatmap shows the relative abundances at the genus level that differed significantly between clusters. C: Control group sample, M: microcapsules treatment group sample, D7 (DSS treatment for 7 days), D15 (DSS treatment for 15 days), V: vancomycin treatment sample.

To further analyze the differences in the fecal bacterial community among all groups, UPGMA clustering tree analysis was used (Fig 10). The differential microorganisms in stool samples for each group (C, M, D15, D7, and V) were analyzed. Our results indicated that vancomycin group samples showed depletion in all prevalent Bacteroidetes and most Firmicutes. Additionally, the abundance of most analyzed genera and OTUs were also significantly changed by vancomycin treatment compared to the control group. We also detected enrichment for multiple *Escherichia*-*Shigella* and *Veillonella* genera in the vancomycin group. While the M group was characterized by the richness of Firmicutes and Bacteroidetes phyla. Furthermore, 31 common genera such as *Streptococcus*, *Lactococcus*, and *Lachnospiraceae* were observed to be increasing in the M group samples.

**Fig 11:**
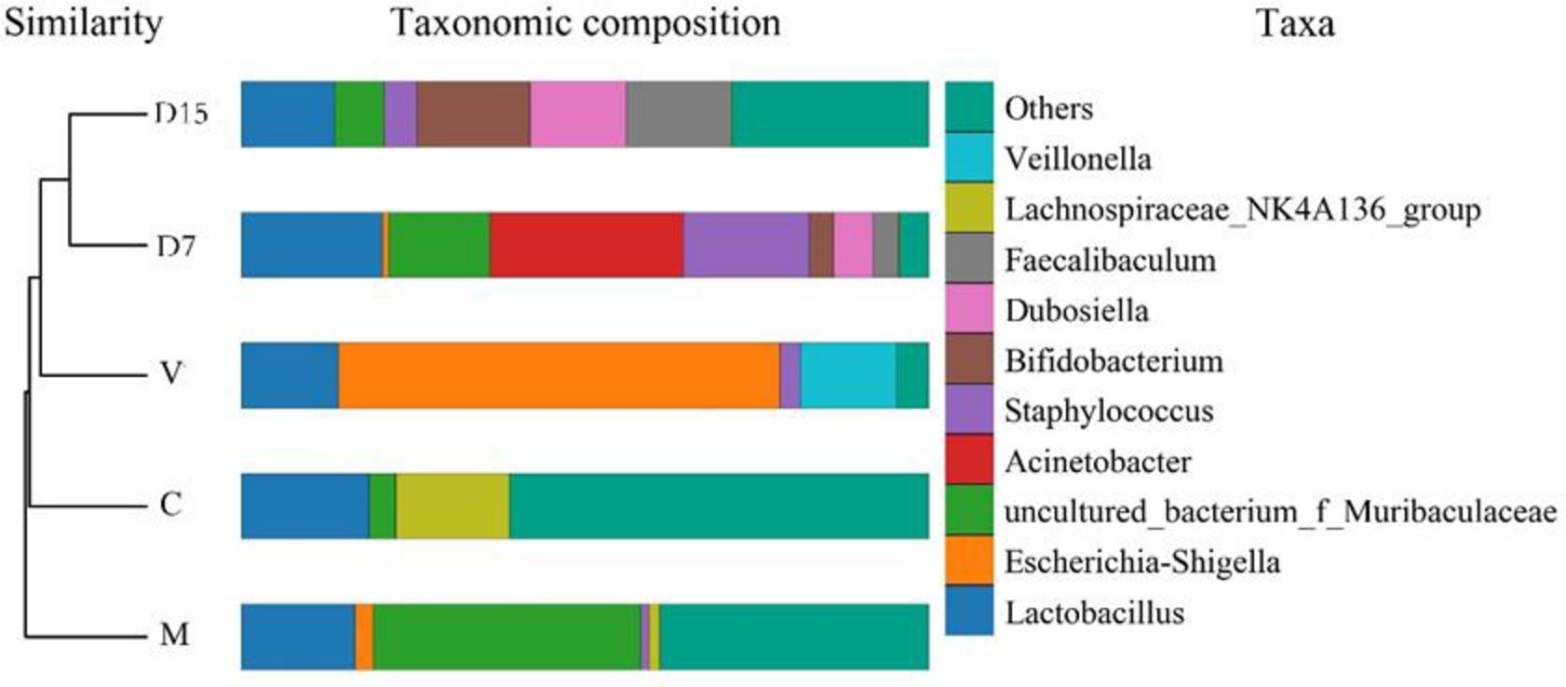
Taxonomic composition distribution of genus-level and UPGMA clustering tree for different treatment groups. Only the top 10 genera are shown for taxonomic composition distribution, and other genera were classified as “other”. In the UPGMA tree, treatment groups started from the D15, D7, V, C, and M are shown as DSS treatment 15 days, DSS treatment 7 days, Vancomycin group, normal group, and Freeze dried microcapsules group, respectively.

The UPGMA clustering tree at the genus level was generated (Fig 11). DSS groups (D15, D7) were clustered together, whereas separated the treatment and control groups from each other, suggesting that these groups had a distinct bacterial community. In the taxonomic composition distribution figure of genus level (Fig 11), the abundances of genera *Escherichia- Shigella* and *Veillonella* of the V group were higher than those of other treatment groups.

## 4. Discussion

UC is a chronic inflammatory disease affecting the colon, and the incidence of the disease has been recently increasing worldwide. The pathogenesis of UC is multifactorial, including genetic factors, dysregulated immune responses, epithelial barrier defects, and environmental factors. Generally, the imbalance of gut microbiota plays a significant role in the development of UC [42–44], maybe due to the abnormal interaction of the gut microbiota with the immune system [2, 3]. Many novel therapeutic strategies have been developed in recent decades to induce endoscopic and clinical remission in UC patients. Antibiotics have proven their ability to inhibit the development of colitis [45, 46]. Experimentally, oral vancomycin is used to evaluate changes during chronic disease models due to the lack of systemic absorption and its localized effects on the gut [47]. In this study, after seven days of treatment, our results indicated that oral vancomycin treatment showed higher efficiency than FMT and LBP freeze-dried powder. Our findings are in agreement with those of another study by Tanaka et al. findings that reported the important role of vancomycin-sensitive bacteria in the severity of colitis [48]. In addition, Rath et al. also reported that vancomycin-imipenem treatment was effective in preventing and treating DSS-induced experimental colitis [49]. Furthermore, meta-analyses of numerous randomized controlled trials, including over 5000 patients, revealed higher total remission rates with antibiotics treatment of active UC [5, 50]. Otherwise, studies that used individual antibiotics such as ciprofloxacin or vancomycin have not proven effective in IBD because the short-term benefits did not translate into longer-term ones [4]. To date, the studies that have evaluated the effectiveness of FMT in UC are limited. However, the first study that used a nasoduodenal tube for FMT showed no significant difference in the endoscopic and clinical remission rate between FMT and control groups [51]. Otherwise, other studies using lower gastrointestinal microbiota transplantation demonstrated encouraging results [31, 32, 37, 52]. However, interesting results were observed two-week after the FMT course [53]. In this study, our discouraged results related to the therapeutic effects of 7 days of FMT and LBP freeze-dried powder treatments were probably due to the short duration of treatment. In this context, Zhang et al. noted that in four patients with UC, three days of treatment with FMT couldn’t achieve clinical improvement, but after three months post-FMT, clinical status improvement was observed [54]. Various strains of probiotics have been used in the IBD treatment, such as *Lactobacillus casei*, *Lactobacillus acidophilus*, etc. [55–57] A study by Chen et al. reported the therapeutic effects of four strains of probiotics in experimental colitis [58]. In addition to probiotics, studies indicated that FMT has also achieved good results in IBD treatment [59–61]. While Wei et al. reported that FMT could alleviate acute colitis induced by DSS in mice [62], other studies pointed out that FMT treatment did not achieve any benefit for patients with UC [51, 63].

In this study, body weight decreased during the administration of the DSS solution, while a significant weight gain was observed during the treatment period. These findings are consistent with previous studies [58, 62, 64, 65]. Generally, inflammatory cytokines, especially IL-1 secreted by mononuclear macrophages, play a crucial role in the early stages of IBD development and experimental colitis, where a high concentration of IL-1, particularly IL-1β, can lead to UC [40]. Our results showed that after seven days of treatment, the level of IL-1β was lower in the vancomycin group than in the other treatment groups. This finding is inconsistent with Tanaka et al. results which indicated that vancomycin could not reduce the level of IL-1β in DSS-induced colitis [48]. However, expanded T cell reaction and the production of significant levels of inflammatory cytokines, such as TNF-α, were noted after the induction of DSS experimental colitis in mice [66]. Our results showed that the level of TNF-α was lower in the vancomycin group than in other treatment groups. This result agrees with Tanaka et al. findings that vancomycin can significantly reduce TNF-α concentration in DSS-induced colitis [48].

To investigate the impact of treatment duration and preparation methods on the effectiveness of microbial therapies (FMT- LBP) in the colitis model, we compared the effect of FMT and LBP microcapsules treatments for 15 days to oral vancomycin treatment on the severity of experimental colitis. Our study indicated that 15 days of LBP microcapsules treatment showed interestingly therapeutic effects in DSS-induced colitis, confirming that the delivery of orally administrated microcapsules may be effective by resisting the harsh environmental conditions within the gastrointestinal tract, including acidic conditions of the stomach, digestive enzymes, and bile salts of the small intestine. The microencapsulation technique seems to be a potential solution to improve the survivability of probiotics [67]. Remarkably, using the microcapsules in polymeric membranes successfully protected viable probiotic bacteria following oral delivery in human and animal models [68]. Several studies have shown discouraged results about the therapeutic effects of probiotic strains on IBD patients, probably because of the short duration of the treatment or the use of inappropriate strains [69]. Zhang et al. showed that the long-term treatment of probiotic strains exerted significant anti-inflammatory effects on DSS experimental colitis [16]. In this study, our results showed that the DAI scores decreased in all treatment groups, especially in the M group. However, no previous studies reported the effect of 15 days of LBP microencapsulated strain treatment on DAI scores or the body weight of mice with experimental colitis. In addition, our results showed that the lowest concentrations of IL-1β and TNF-α were observed in the LBP microcapsules treatment group.

The balance of gut microbiota is closely relevant to human diseases and health [70]. Here, we investigated the effect of LBP microcapsules and vancomycin on the fecal microbial diversity in the DSS-induced colitis model. The oral vancomycin treatment has shown efficacy in UC patients due to its effect on gram-positive bacteria in the gut microbiota [71]. Our results have shown that vancomycin depletes most bacterial OTUs found in the feces microbiota, including all detected baseline OTUs from the phylum Bacteroidetes. In addition, after vancomycin treatment, increasing the abundance of members belonging to the phylum Proteobacteria, which are usually related to human infections such as *Escherichia* and *Shigella*, was observed. Our results are consistent with several previous studies. Isaac et al. have compared the effect of long-and short-term oral vancomycin treatment on the gut microbiota and reported that vancomycin therapy caused depletion the most of the intestinal microbiota genera and OTUs, especially members of phylum Bacteroidetes, in addition to increasing the susceptibility to intestinal pathogens [72]. Another study by Sun et al. reported that vancomycin treatment significantly increased the abundance of the Proteobacteria phylum members, altered the microbial composition at phylum and family levels, and decreased richness and diversity at the species level [73]. In addition, Tanaka et al. indicated that oral vancomycin therapy reduced the number of gram-positive and anaerobic gram-negative bacteria, which explains our results that OTUs numbers of the vancomycin groups were lower than other groups. Because vancomycin-sensitive bacteria attract neutrophils to the damaged colon tissue that induce inflammation and DNA damage in the colon [48], our results suggested that vancomycin treatment reduced colon inflammation in DSS-induced colitis in short-term treatment experiments by reducing the vancomycin-sensitive bacteria. On the other hand, our results indicated that LBP microcapsules restored most bacterial OTUs found in the fecal microbiota compared to the control and DSS groups. Microcapsules treatment increased the microbial diversity and richness compared to vancomycin treatment and reduced the relative abundance of Lactobacillus compared to the control group. These findings are in agreement with Zhang et al. study, which investigated the impacts of *Lactobacillus plantarum* on colonic bacteria in DSS-induced colitis and reported that the *L*.*plantarum* treatment increased the diversity of colonic bacterial but reduced the relative abundance of Lactobacillus [74]. Significantly, our high-throughput sequencing results defined all prevalent OTUs belonging to the Bacteroidetes phylum affected by vancomycin treatment. Consistent with our findings, Vrieze et al. also reported a decrease in the levels of Ruminococcus and Faecalibacterium after oral vancomycin treatment [75]. Our results indicated that the microbial structure in the LBP microcapsules treatment group was characterized by dominated of Muribaculaceae. Muribaculaceae is one of the short-chain fatty acids (SCFAs)-producing bacteria [76]. Smith et al. have also reported the correlation between the increasing total SCFAs and the increased relative abundance of Muribaculaceae in mice feces [77]. Recent studies have shown that SCFAs affect the clinical progression of IBD due to their significant anti-inflammatory functions regulating immune function and preventing an excessive immune response [78, 79]. In this study, our results suggested that the LBP microcapsules treatment improved the inflammatory response in DSS-induced acute colitis after 15 days of treatment.

Altogether, our results indicated the negative consequences of oral administration of vancomycin that must be considered before prescribing this antibiotic. In addition, this study suggested that LBP microcapsules have shown promising results on the fecal microbial diversity and species richness in DSS-induced colitis in mice.

## 5. Conclusion

UC is a subtype of chronic IBD of unknown etiology that makes finding appropriate treatment a major challenge. Although gut microbiota plays a significant role in UC development, most available therapies target the immune response. In this study, we compared the efficacy of oral vancomycin and two different microbial strategies in experimental colitis treatment. Our results showed that vancomycin therapy reduced the severity of experimental colitis but induced Bacteroidetes depletion and concomitant expansion of Proteobacteria. Whereas LBP microencapsulation therapy was superior to vancomycin due to the reduction of colitis severity, restoration of fecal bacterial composition, and increase of fecal microbial diversity and richness.
